# Trends and Patterns in Wealth Index Assets: Observations From 68 Countries

**DOI:** 10.9745/GHSP-D-24-00132

**Published:** 2026-08-10

**Authors:** Andrew Corley, Claire Dunn, Yuen Wai Hung, Andrea Sprockett, Dominic Montagu, Nirali Chakraborty

**Affiliations:** a Metrics for Management, Baltimore, MD, USA.

## Abstract

This commentary outlines 4 practical lessons derived from analyzing a country-specific measure of wealth available for use in 68 countries, serving as axioms for development practitioners to simplify questions related to identifying relative poverty and project design and implementation.

## Key Messages

- This commentary outlines 4 practical lessons derived from analyzing a country-specific measure of wealth available for use in 68 countries, serving as axioms, or rules of thumb, for development practitioners to simplify questions related to identifying relative poverty and for informing project design and implementation.
- Dwelling building materials strongly indicate relative wealth or poverty.
- Ownership of consumer electronics, appliances, and cleaner cooking fuels inside homes correlates with wealth.
- The assets that best indicate relative wealth vary based on a country’s economic development level.
- Assets indicating wealth may change over time due to affordability and technological advancements, with certain assets losing their discriminatory power while others gain significance.

## INTRODUCTION

Human development indicators and socioeconomics are tightly intertwined. Improved outcomes related to issues as diverse as educational attainment, tuberculosis treatment, and estimations of happiness and life satisfaction have all been associated with higher socioeconomic status.^1^^–^^4^ It is no surprise then that reaching those at the lowest ends of the economic spectrum has been identified as key to achieving many of the United Nations’ Sustainable Development Goals.^5^

While the importance of reaching the poor has been identified as key to overall development, measuring socioeconomic status in an accurate and timely manner to allow for programmatic action has historically been a challenge. Two frequently used proxy indicators of socioeconomic status—income and consumption—are generally unable to provide timely and accurate measurement, particularly in low- and middle-income countries. Income is particularly difficult to capture accurately as respondents are hesitant to report their incomes when surveyed and, even if reported, incomes are often poor representations of wealth due to frequent month-to-month or seasonal fluctuations, especially among the poorest.^6^ Consumption indicators are also quite difficult to collect, requiring burdensome data collection efforts, and concerns remain about what consumption items should be included and over what time period.^6^

Due to these shortcomings, scholars and practitioners have sought alternative measures of socioeconomic status. One alternative that emerged in the early 2000s was the asset or wealth index. Emerging research suggested that applying dimensionality reduction statistical methods to household characteristics and asset ownership data could produce reliable proxies of household wealth. When implemented, the first wealth indices proved easier to estimate than income- and consumption-derived measures of socioeconomic status.^7^ Facilitated by their adoption by the Demographic and Health Surveys (DHS) Program’s suite of surveys, the Multiple Indicator Cluster Surveys (MICS) program, and other related household surveys, wealth indices have become a standard means of measuring equity in health and other development fields, such as water and sanitation, social protection, and education.

While collecting the underlying data of a wealth index is often simpler than alternative measures of socioeconomic status, the typical wealth index often requires answers to 25 to 50 multiple-choice questions, some of which can be challenging for respondents to answer on their own without a trained interviewer present.^8^ The EquityTool (www.equitytool.org) was designed to offer a simplified approach to measuring relative wealth backed by rigorous evidence.

Following a process developed by a consortium of leading experts in wealth measurement, the EquityTool prioritizes assets that are most able to differentiate households based on the latent concept of wealth.^9^ An EquityTool for a country is developed by first ranking the variables used in the full wealth index of a recent nationally representative household survey, such as the DHS or MICS, according to their statistical importance. Variables from the full wealth index are then sequentially added to the EquityTool questionnaire based on their statistical significance. This process continues until the EquityTool simplified index achieves a sufficient level of agreement with the full wealth index, indicated by a kappa statistic (k) of 0.75 or higher. This process results in a short, country-specific questionnaire that can be used to assess the relative wealth of populations in an average of 12 questions.

The EquityTool was created with global health and development practitioners in mind. Since it can be challenging to ask respondents numerous questions about their households when they are out and about at a market or health clinic, EquityTools are designed to be as short as possible while still yielding valid relative wealth distributions. Their questions are also designed to be easy for respondents to answer. For example, while wealth indices include questions with continuous response options, such as the number of acres a household uses for agriculture or the number of heads of various livestock they own, EquityTools only use questions with dichotomous yes/no responses, such as whether any household member owns a watch. This reduces the cognitive burden on respondents by relieving them of having to reflect on more nuanced questions. Like the DHS and MICS, the EquityTool is constructed using households as units and then applied to all individuals in the household.

Metrics for Management (M4M) began producing the EquityTool in 2015 and has since produced national-level EquityTools for 70 countries, updating the tool when new wealth index surveys become available. Since then, the EquityTool has been widely adopted by practitioners working in health and development.^10^^–^^15^ In producing the EquityTool, M4M has gleaned several lessons that are useful for practitioners who want to target their programs to the poor. Analyzing the assets that appear across different versions of the EquityTool can prove particularly informative given that the EquityTool offers us the ability to explore asset patterns across an array of different household survey types beginning from a reduced set of variables that have been identified for their strong ability to differentiate between wealth quintiles. Instead of employing the EquityTool as a metric for relative wealth, in this commentary it serves as a lens through which to extract broader insights about identifying indicators of wealth. This shift in application allows us to explore some of the fundamental elements of economic and human development.

In the remainder of this article, we outline 4 lessons we have learned from analyzing the 98 EquityTools produced to date. These lessons can serve as practical rules of thumb for inexperienced and experienced development and global health practitioners alike. For students and those newer to the discipline, these lessons can serve to simplify complex development questions, reduce cognitive load, and accelerate learning. For those experienced practitioners who already intuitively understand that ownership of certain assets or durable goods are signifiers of wealth, this article empirically identifies those assets that strongly differentiate relatively poor households from relatively wealthy ones. This information will arm all readers with an evidence-based starting point for project design and implementation. Using these guides, practitioners can narrow in on potential intervention designs and implementation sites before investing additional time and resources in wealth measurement to confirm fit.

## ANALYSIS OF EQUITYTOOL ASSETS

**FIGURE 1:**
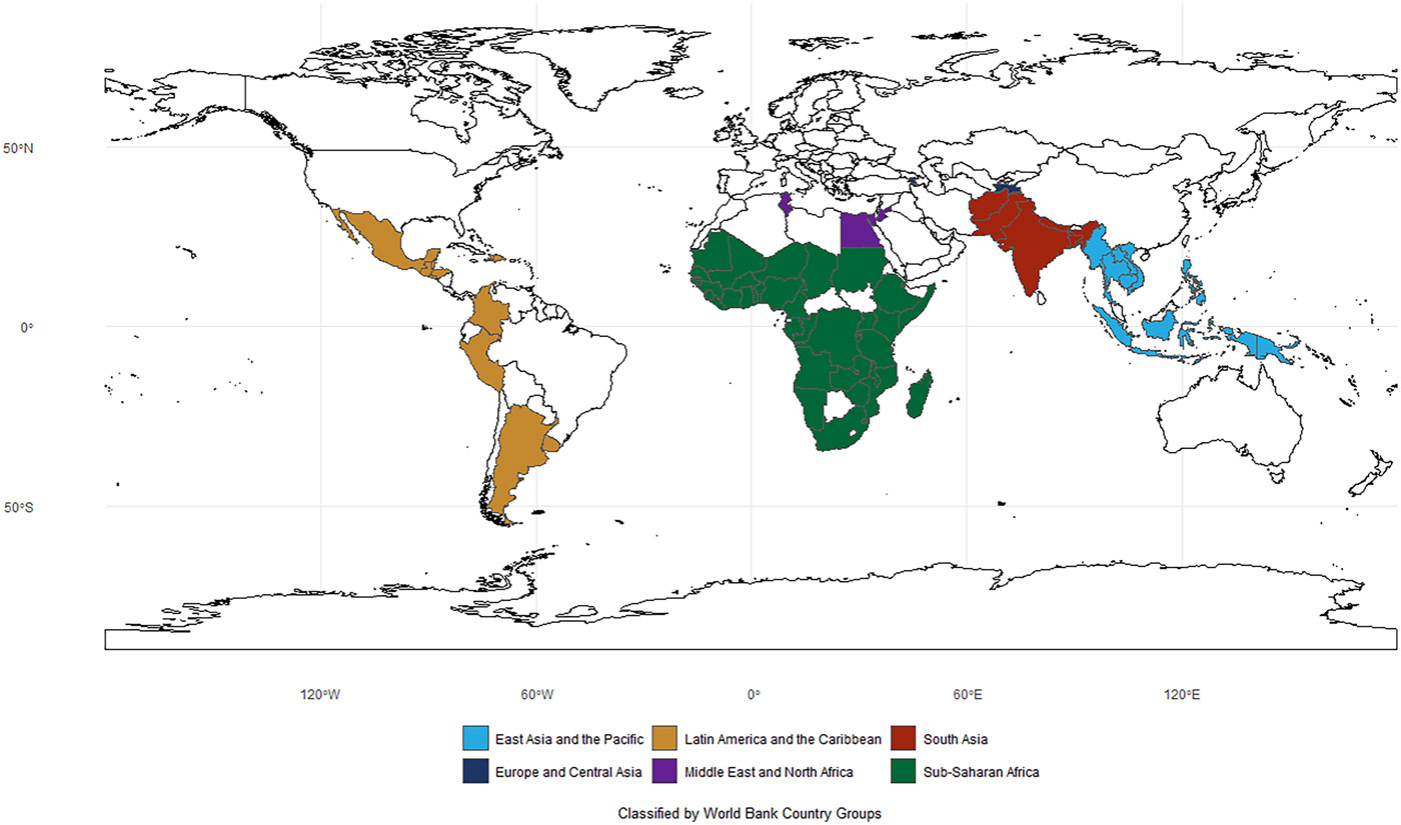
Geographic Distribution of EquityTool Countries

The lessons presented here are derived from our analysis of the assets that appear in the 98 EquityTools creating by M4M between 2015 and 2023, covering 68 countries and 12 years of surveys (2009–2021). Countries’ EquityTools are updated as new household survey data becomes available, leading to more EquityTools than countries. Table 1 lists the countries and their regional categories, and Figure 1 illustrates their geographic distribution.

**TABLE 1.** EquityTool Countries by Region

Region	No. of Countries	Countries
East Asia and the Pacific	9	Cambodia, Indonesia, Laos, Myanmar, Papua New Guinea, Philippines, Thailand, Timor Leste, Vietnam
Europe and Central Asia	2	Armenia, Tajikistan
Latin America and the Caribbean	11	Argentina, Belize, Colombia, Dominican Republic, El Salvador, Guatemala, Haiti, Honduras, Mexico, Peru, Uruguay
Middle East and North Africa	3	Egypt, Jordan, Tunisia
South Asia	5	Afghanistan, Bangladesh, India, Nepal, Pakistan
Sub-Saharan Africa	38	Angola, Benin, Burkina Faso, Burundi, Cameroon, Chad, Côte d’Ivoire, Democratic Republic of the Congo, Djibouti, Eswatini, Ethiopia, Gabon, Gambia, Ghana, Guinea, Guinea-Bissau, Kenya, Liberia, Madagascar, Malawi, Mali, Mauritania, Mozambique, Namibia, Niger, Nigeria, Republic of Congo, Rwanda, Senegal, Sierra Leone, Somalia, South Africa, Sudan, Tanzania, Togo, Uganda, Zambia, Zimbabwe

When creating the EquityTool, the wealth index from the original household survey, often the DHS or MICS, serves as the starting point. These full wealth indices use principal components analysis to assign weights to each asset based on their contribution to the underlying concept of wealth. To create the EquityTool, assets are sorted based on their statistical importance to the full wealth index, measured by multiplying the absolute value of the factor weight of the first principal component by the standard deviation of that variable. Variables are added to the EquityTool based on their importance until both the national and urban EquityTool indices reach a level of agreement with the original wealth index where kappa is at least 0.75. Once the most important assets have been identified, scores are assigned to each asset in line with the scores from the full wealth index and wealth quintiles are determined based on the household’s total score. This approach results in a shortened but reliable index that includes only those assets that are most important for differentiating between wealth quintiles in each country. By focusing on assets that appear in an EquityTool in this analysis, we are able to consider assets that appear in a variety of different household surveys as well as focus the analysis on a smaller set of assets that have already been identified as having the strongest ability to differentiate between wealth quintiles in each country.

In total we identified 99 assets across these 98 EquityTools. From this compiled list of assets, we counted the frequency of appearance of each asset. In order to draw more general conclusions about the types of assets that are frequently found to be good classifiers of household wealth, we also categorized the assets into broader asset categories. For example, while an air conditioner may appear in some wealth indices, a space heater may appear in others, but both of these assets can be categorized as appliances. We used the standard asset categories found in the DHS Phase 8 core questionnaire for assets, such as dwelling construction materials, cooking fuels, and toilet facility types, as a starting point for the creation of our own categories. In total we identified 23 asset categories (see Supplement) and counted the frequency of appearance of these broader asset categories among the EquityTools.

In order to assess the extent to which assets vary in importance over time, we divided our sample of EquityTools into two groups. The first group includes EquityTools developed using household surveys released between 2009 and 2014. The second group includes EquityTools developed using surveys released between 2015 and 2021. This results in a fairly even division of EquityTools between the two time periods. There is no significant difference in the relative proportions of low-, lower middle-, and upper middle-income countries included in the two groups.

From these analyses, we identified 4 general rules, or axioms, which can be used to conduct rapid initial assessment of wealth.

## FOUR AXIOMS FOR RAPID WEALTH ASSESSMENTS

### Axiom 1: Assets Vary by Economic Development Classification

A country’s level of economic development strongly influences the types of assets that are most appropriate for classifying its households’ relative wealth. The asset ownership profile of the typical household in the third quintile of a low-income country looks significantly different from that of a typical household in the same quintile in an upper-middle-income country.

Table 2 helps to illustrate this point. When stratified by World Bank income groups, we see that most EquityTools for low-income and lower-middle-income countries ask about ownership of a bank account and access to electricity. Access to basic financial services and electrification are fundamental to economic development and, in the case of electricity, a necessary precursor to the acquisition of many other assets, such as appliances and consumer electronics. In more developed regions, however, access to essential financial services and electrification is nearly universal, and so these assets lose their ability to discriminate between wealth quintiles. To differentiate between wealth quintiles in high-income countries, the focus must shift to other forms of infrastructure. Within the range of assets that can be considered as infrastructure, we see that internet access is a more prevalent infrastructure asset question in upper-middle-income countries’ EquityTools than in other country income groups. Further, with the ready availability of electricity and increasing importance of internet access, we also observe a corresponding increase in the prevalence of computer ownership as a question posed in EquityTools in lower- and upper-middle-income countries.

**TABLE 2.** Frequency of Appearance of Assets in EquityTools by Country Income Group,^a^ No. (%)

Asset	Low-Income Countries (N=26)	Lower-Middle-Income Countries (N=31)	Upper-Middle-Income Countries (N=11)
Bank account	19 (73.1)	22 (71.0)	—
Bed	7 (26.9)	—	—
Car or truck	—	—	5 (45.5)
Chair	7 (26.9)	—	—
Computer	—	18 (58.1)	8 (72.7)
Cooking fuel	23 (88.5)	24 (77.4)	3 (27.3)
Cupboard/bookcase	7 (26.9)	—	—
Drinking water source	11 (42.3)	14 (45.2)	5 (45.5)
DVD/VCD player	—	—	3 (27.3)
Electric fan	—	12 (38.7)	—
Electricity	23 (88.5)	12 (38.7)	—
Exterior wall material	19 (73.1)	16 (51.6)	4 (36.4)
Floor material	24 (92.3)	22 (71.0)	6 (54.5)
Internet access	—	—	4 (36.4)
Landline telephone	—	—	4 (36.4)
Microwave	—	—	7 (63.6)
Mobile phone	9 (34.6)	—	—
Radio	13 (50.0)	—	—
Refrigerator	12 (46.2)	27 (87.1)	5 (45.5)
Roof material	19 (73.1)	14 (45.2)	6 (54.5)
Sofa/divan	—	—	5 (45.5)
Television	22 (84.6)	19 (61.3)	—
Toilet facility	14 (53.8)	19 (61.3)	9 (81.8)
Washing machine	—	11 (35.5)	5 (45.5)
Watch	13 (50.0)	10 (32.3)	—

^a^ Contains all assets appearing in 25% or more of the most recent EquityTool for each country for any of the 3 country income groups.

Additionally, while some asset categories are present across all country income groups, the subtypes of these assets vary by countries’ income. This can be seen in the categories of flooring, wall, and roof materials (Table 3); cooking fuels (Table 4); water source (Table 5); and toilet type (Table 6). All these asset categories are salient features of EquityTools of countries across the wealth spectrum, but in all these categories we observe a shift away from questions related to having rudimentary versions of these asset categories to questions related to possessing more finished versions. Among the EquityTools for upper-middle-income countries, we see that rudimentary and natural building materials for flooring, roofing, or walls no longer appear. Rather, questions focus on the type of finished materials used, reflecting the fact that a larger portion of the population can afford improved housing.

**TABLE 3.** Frequency of Appearance of Type of Roof, Wall, and Floor Materials in EquityTools by Country Income Group,^a^ No. (%)

Material Type	Low-Income Countries (N=43)			Lower-Middle-Income Countries (N=43)			Upper-Middle-Income Countries (N=12)		
	**Roof**	**Wall**	**Floor**	**Roof**	**Wall**	**Floor**	**Roof**	**Wall**	**Floor**
Finished	28 (65.1)	29 (67.4)	33 (76.7)	18 (41.9)	26 (60.5)	21 (48.8)	9 (75.0)	12 (100.0)	9 (75.0)
Rudimentary	2 (4.7)	1 (2.3)	8 (18.6)	2 (4.7)	4 (9.3)	1 (2.3)	—	—	—
Natural	23 (53.5)	40 (93.0)	16 (37.2)	4 (9.3)	20 (46.5)	10 (23.3)	—	—	—

^a^ Contains data from all EquityTools. Some EquityTools include more than one asset item.

**TABLE 4.** Frequency of Appearance of Cooking Fuels in EquityTools by Country Income Group,^a^ No. (%)

Cooking Fuel	Low-Income Countries (N=43)	Lower-Middle-Income Countries (N=43)	Upper-Middle-Income Countries (N=12)
Agricultural products (crops, straw, shrubs, grass)	2 (4.7)	2 (4.7)	—
Animal dung	1 (2.3)	—	—
Charcoal	18 (41.9)	2 (4.7)	1 (8.3)
Electricity	4 (9.3)	4 (9.3)	1 (8.3)
Kerosene	—	1 (2.3)	—
Cooking gas (LPG, natural, biogas)	4 (9.3)	24 (55.8)	3 (25.0)
Wood	33 (76.7)	24 (55.8)	1 (8.3)

Abbreviation: LPG, liquefied petroleum gas.

^a^ Contains data from all EquityTools. Some EquityTools contain more than one cooking fuel asset question.

**TABLE 5.** Frequency of Appearance of Water Sources in EquityTools by Country Income Group,^a^ No. (%)

Water Sources	Low-Income Countries (N=43)	Lower-Middle-Income Countries (N=43)	Upper-Middle-Income Countries (N=12)
**Piped water**			
Piped into dwelling	8 (18.6)	8 (18.6)	4 (33.3)
Piped into a yard/plot	6 (14.0)	—	—
Public tap/standpipe	3 (7.0)	—	—
Tube well or borehole	1 (2.3)	1 (2.3)	—
**Packaged/sold water**			
Bottled water	—	7 (16.3)	1 (8.3)
Sachet water	—	2 (4.7)	—
Water sales company	2 (4.7)	—	—
**Protected ground source**			
Protected spring	—	1 (2.3)	—
Protected well	1 (2.3)	1 (2.3)	—
**Unprotected ground or surface source**			
Surface water (river/dam/lake/pond/stream/canal/irrigation channel)	1 (2.3)	1 (2.3)	—
Unprotected spring	4 (9.3)	—	—
Unprotected well	3 (7.0)	2 (4.7)	—

^a^ Contains data from all EquityTools. Some EquityTools contain more than one water source asset item.

**TABLE 6.** Frequency of Toilet Types in EquityTools by Country Income Group,^a^ No. (%)

Toilet Types	Low-Income Countries (N=43)	Lower-Middle-Income Countries (N=43)	Upper-Middle-Income Countries (N=12)
**Flush or pour flush toilet**			
Flush to the piped sewer system	6 (14.0)	10 (23.3)	9 (75.0)
Flush to pit latrine	1 (2.3)	—	—
Flush to septic tank	11 (25.6)	10 (23.3)	1 (8.3)
Flush to somewhere else	1 (2.3)	—	1 (8.3)
Toilet in own dwelling	—	3 (7.0)	2 (16.7)
Toilet outside dwelling	—	—	1 (8.3)
**Pit latrine**			
Pit latrine with slab	7 (16.3)	—	—
Pit latrine without slab/open pit	4 (9.3)	4 (9.3)	—
Ventilated improved pit latrine	1 (2.3)	—	—
**No facility/bush/field**			
No facilities/bush/field	15 (34.9)	7 (16.3)	—
**Other**			
Any type of toilet/latrine	—	1 (2.3)	1 (8.3)

^a^ Contains data from all EquityTools. Some EquityTools contain more than one toilet type asset item.

### Axiom 2: Appearances Matter

Before conducting a single interview, you can learn a lot about the relative wealth or poverty of a community simply by observing your surroundings. Our analyses show that much can be gleaned from observing the built environment, in particular. Building materials, including floor, roof, and wall materials, are routinely included in EquityTools, making up 3 of the top 10 most frequently appearing assets, with the added benefit of being consistent predictors of either wealth or poverty. This means that someone whose home is made of more finished materials will almost always be classified as being relatively wealthier than someone whose home is constructed of more rudimentary materials, across urban and rural contexts and countries. For example, the presence of natural building materials, such as earth or sand floors, thatch roofs, or dirt walls are almost always indicative of poverty, whereas finished materials such as ceramic tile floors, concrete roofs, or brick walls suggest relatively wealthier households (Table 3). The positive correlation between more finished materials and wealth stands in contrast to other assets that can indicate either wealth or poverty depending on the context. For instance, ownership of livestock in rural areas frequently suggests greater wealth, while in urban settings ownership of these same assets frequently indicates greater relative poverty.

The importance of building materials is consistent regardless of whether the country is a low-, lower-middle-, or upper-middle-income country. In each of the country income groups, building materials appear in at least 40% of all EquityTools.

### Axiom 3: Wires, Warmth, and Water

**FIGURE 2:**
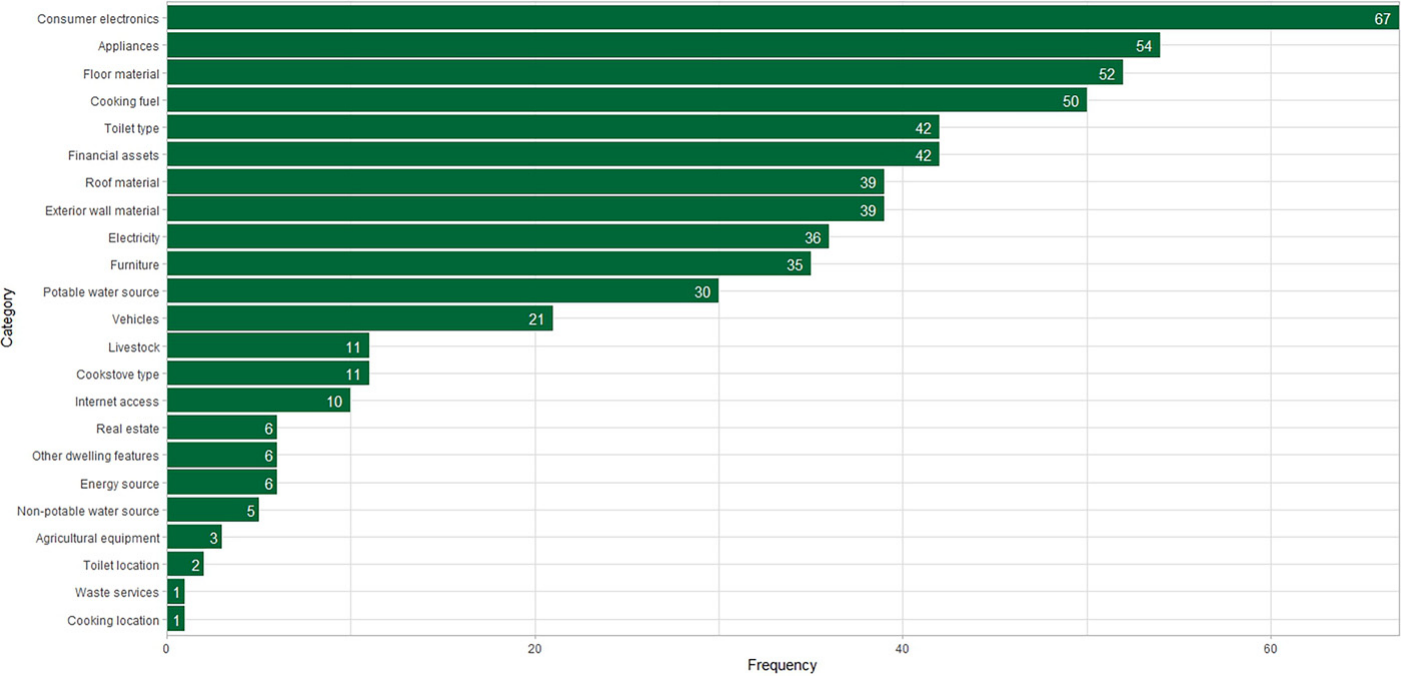
Frequency of Appearance of Unique Asset Categories in Most Recent EquityTools (N=68)

When looking globally, we see in Figure 2 that nearly every country’s most recent EquityTool (67 of 68) included at least one question about ownership of consumer electronics. Similarly, 54 of 68 of the most recent EquityTools include at least one question about ownership of an appliance. There are a number of different consumer electronics and appliance assets (see Supplement); however, television is the most frequently cited, appearing in approximately 63% of EquityTools. Refrigerator ownership, the most frequently cited appliance asset, appears in approximately 65% of all EquityTools.

The type of cooking fuel a household uses is another important asset class found inside homes that is similarly predictive of relative wealth (Table 4). Cleaner and more efficient cooking fuels such as electricity, liquefied petroleum gas (LPG), and natural gas are suggestive of higher relative wealth, while solid fuels such as wood, charcoal, and agricultural products suggest lower relative wealth. There are important economic and environmental implications of different cooking fuel choices, with cleaner fuels being associated with higher economic development and lower environmental impact.^16^^,^^17^ Importantly, cleaner, more efficient forms of cooking fuel are not only a sign of greater relative wealth but can also serve to create these conditions. Cleaner cooking fuels, such as electricity or LPG, can significantly reduce the time spent on fuel collection and cooking activities. These time savings allow households, particularly women and girls who are often responsible for cooking and solid fuel collection, to reallocate their time and energy toward education and other economically productive endeavors. Moreover, the adoption of cleaner cooking fuels can lead to improved health outcomes, reducing the economic burden of health care costs and enhancing overall well-being.^18^^,^^19^

Finally, much can be learned about a household’s relative wealth from their source of drinking water and their toilet facilities. These key aspects of water, sanitation, and hygiene (WASH) are among the top 10 most frequently appearing assets in all EquityTools and are useful for determining wealth in countries at all levels of economic development. Drinking water sources appear in about 40% of the 68 EquityTool countries’ most recent tools (Table 5), while toilet facilities appear in nearly 62% of these same EquityTools (Table 6). Access to safe drinking water and improved toilet facilities are not only symbols of wealth but are also associated with reduced prevalence of infectious diseases and improved economic outcomes.^20^

### Axiom 4: The Assets That Tell Us About Wealth Can Change Over Time

The ability of assets to distinguish between wealth quintiles can change over time for a variety of reasons. Some assets may become relatively more affordable over time, making them accessible to households that previously could not have purchased them. Alternatively, technological change may lead to the introduction of new assets and reduce the relevance of others.

**FIGURE 3:**
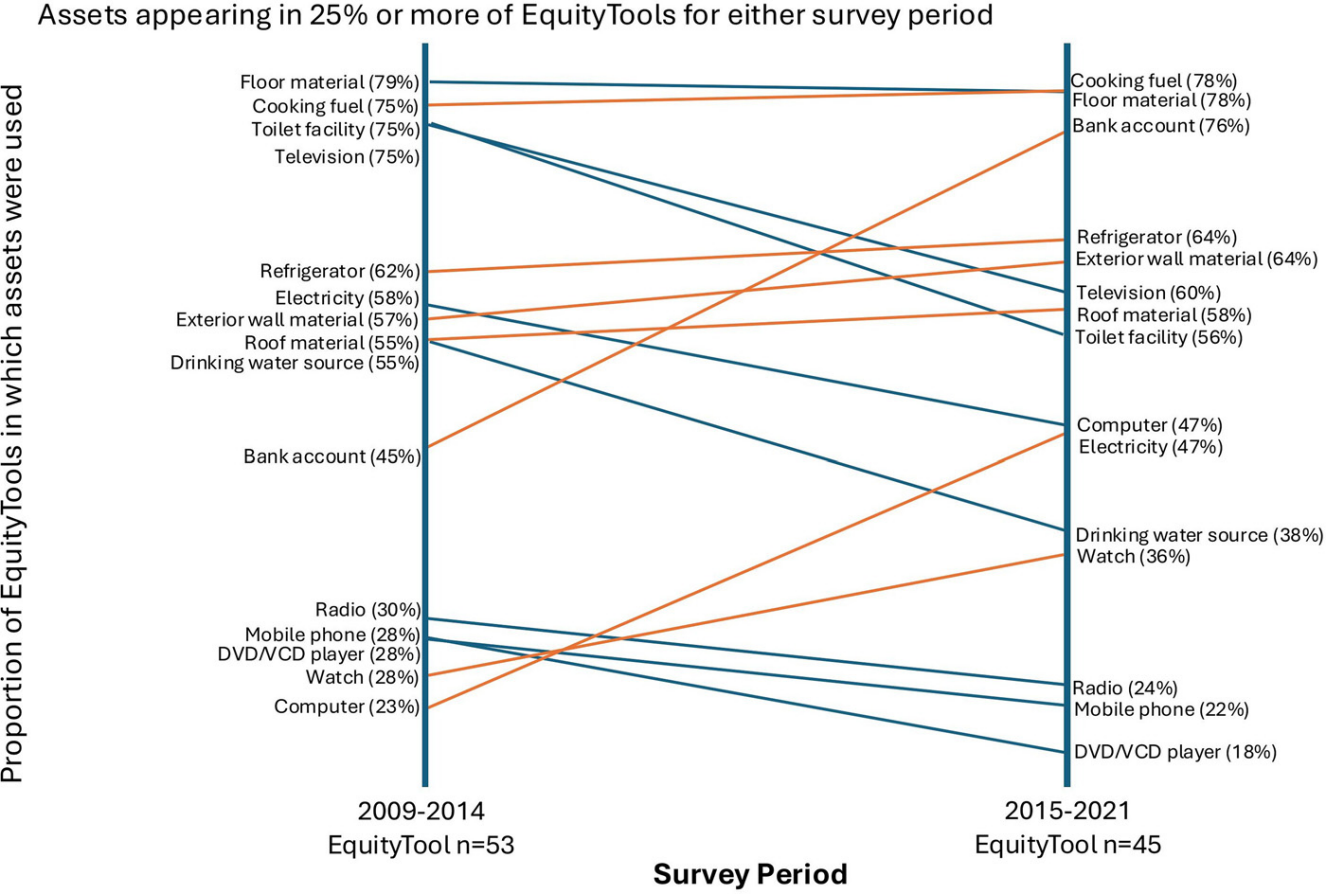
Changes in EquityTool Asset Appearance Over Time

Though we have ensured that there is no significant difference between the distribution of low-, lower-middle, and upper-middle-income countries in each of the two survey periods of 2009–2014 and 2015–2021 (χ2 (4, *N* = 98) = 6; *P* = .20), much of the change in assets’ prevalence in EquityTools between the two time periods can be attributed to variance in the data (Figure 3). However, there are still some observations worth making. The first of these is that building materials remain important factors for determining relative wealth over time. The appearance of flooring material in EquityTools fell only 1 percentage point between the earlier and later survey periods, while exterior wall and roof materials increased in prevalence by 7 points and 3 points, respectively. Finished building materials tend to be more expensive than rudimentary materials, but they can help to increase a building’s comfort and long-term structural resilience, making them an enduring marker of a household’s affluence and social standing.

Another interesting observation is the increasing prevalence of questions related to bank account and computer ownership. Having a bank account may be insufficient in itself to alleviate household poverty, but ownership of an account has become an increasingly useful proxy for a household’s relative wealth.^21^ Similarly, the increasing affordability of personal computers over the past decade has allowed this asset to reach an ownership rate threshold that allows it to differentiate between households in the middle and top wealth quintiles.

While bank accounts and computers have grown in their ability to predict household wealth, other assets have seen notable declines in their ability to do so. Mobile phones are one example. Once reserved for only the wealthiest, mobile phone ownership rates have seen significant growth globally over the past two decades.^22^ While mobile phones have become indispensable to the way many individuals communicate and do business, their ubiquity reduces the asset’s ability to discriminate between wealth quintiles. Smartphone ownership may soon become a useful indicator of relative household wealth.^23^

Television ownership is another asset question that has declined in prevalence over time. While still among the most common assets included in EquityTools, questions regarding television ownership have also fallen notably. Though less dramatic than mobile phones, television ownership has grown steadily over time.^21^ Where television ownership is no longer rare, it becomes less useful for distinguishing between wealth quintiles.

Lastly, the decline in questions related to households’ toilet facilities is one trend that cannot be easily explained. Between the 2009–2014 and 2015–2021 periods, the number of EquityTools including questions about a household’s toilet facility dropped by 19 percentage points. Globally, an estimated 2.3 billion people lack access to improved sanitation facilities, and poor sanitation remains a significant determinant of the world’s disease burden.^24^ Proper sanitation facilities remain foundational to disease prevention and are a critical focus area for human development and public health.

## LIMITATIONS

An important limitation of this analysis is that our analyses only include those dichotomous asset variables that were included in EquityTools. Continuous asset variables, such as the number of household members per sleeping room, can be important predictors of relative wealth but are not always easily quantified and thus are not included in EquityTools. Excluding these assets is a key tradeoff of the EquityTool that contributes to its usability. However, since EquityTools prioritize high-information asset variables that can strongly distinguish between wealth quintiles, we believe these assets remain a strong basis for our axioms.

In addition, the guidelines presented here are based only on the EquityTools that have been produced to date. While the EquityTool covers 68 countries, its geographic coverage is limited to those countries in which nationally representative household surveys, such as DHS or MICS, have been conducted. Similarly, updated DHS or MICS surveys that are released provide opportunities for existing EquityTools to be updated. As such, while we believe the findings from this analysis apply to a wide range of contexts, they cannot be considered global.

## CONCLUSION

The construct of wealth, as measured by wealth indices, is an important determinant of many health and developmental issues. At the outset of identifying communities for development and global health interventions, resources for extensive data collection to measure relative wealth may be limited. In such circumstances, employing the guidance presented here is particularly valuable. The axioms in this article provide a practical and cost-effective approach to decision-making when formal measures of wealth are not immediately feasible. Accurately identifying relatively poor households is a critical step that global health and development practitioners must undertake to ensure that their policies and programs reach and maximally benefit their intended beneficiaries.

It is important to keep in mind that the guidance summarized as axioms here are generalizations and that they cannot replace local knowledge. It is thus crucial to conduct assessments in collaboration with local organizations and stakeholders who possess a deep understanding of the local context.

Additionally, since assessment findings represent a snapshot of a dynamic environment, regular evaluations of relative wealth are necessary to capture the evolving nature of socioeconomic conditions, measure the impact of interventions, adapt policies and programs, and ensure ongoing community engagement and empowerment. The results of these assessments are not static; they are influenced by a myriad of factors, including economic, social, environmental, technological, health, and cultural changes, all of which shape the continuously shifting landscape of wealth and development.

Finally, no set of generalized lessons should replace the measurement of socioeconomic status through a validated measure of wealth, such as the EquityTool. However, we believe that the trends and observations presented in this article will help guide practitioners’ thinking and decision-making when working in their communities.

## Notes

**Peer Reviewed**

**First Published Online:** June 18, 2026.
